# Empathy in schizophrenia: neural alterations during emotion recognition and affective sharing

**DOI:** 10.3389/fpsyt.2024.1288028

**Published:** 2024-05-24

**Authors:** Simon Knobloch, Delia Leiding, Lisa Wagels, Christina Regenbogen, Thilo Kellermann, Klaus Mathiak, Frank Schneider, Birgit Derntl, Ute Habel

**Affiliations:** ^1^ Department of Psychiatry, Psychotherapy and Psychosomatics, Faculty of Medicine, Rheinisch-Westfälische Technische Hochschule (RWTH) Aachen University, Aachen, Germany; ^2^ Institute of Systems Neuroscience, Center for Experimental Medicine, University Medical Center Hamburg-Eppendorf (UKE), Hamburg, Germany; ^3^ Psychiatry Neuroimaging Branch (PNB), Department of Psychiatry and Psychotherapy, University Medical Center Hamburg-Eppendorf (UKE), Hamburg, Germany; ^4^ Jülich-Aachen Research Alliance (JARA) – Translational Brain Medicine, Aachen, Germany; ^5^ Department of the History, Philosophy and Ethics of Medicine, Centre for Health and Society (chs), School of Medicine, Heinrich-Heine-University Düsseldorf, Düsseldorf, Germany; ^6^ Department of Psychiatry and Psychotherapy, Tübingen Center for Mental Health (TüCMH), University of Tübingen, Tübingen, Germany; ^7^ German Center for Mental Health (DZPG), Partner Site Tübingen, Tübingen, Germany

**Keywords:** schizophrenia, social cognition, emotion recognition, affective sharing, empathy, fMRI

## Abstract

**Introduction:**

Deficits in emotion recognition and processing are characteristic for patients with schizophrenia [SCZ].

**Methods:**

We targeted both emotion recognition and affective sharing, one in static and one in dynamic facial stimuli, during functional magnetic resonance imaging [fMRI] in 22 SCZ patients and 22 matched healthy controls [HC]. Current symptomatology and cognitive deficits were assessed as potential influencing factors.

**Results:**

Behaviorally, patients only showed a prolonged response time in age-discrimination trials. For emotion-processing trials, patients showed a difference in neural response, without an observable behavioral correlate. During emotion and age recognition in static stimuli, a reduced activation of the bilateral anterior cingulate cortex [ACC] and the right anterior insula [AI] emerged. In the affective sharing task, patients showed a reduced activation in the left and right caudate nucleus, right AI and inferior frontal gyrus [IFG], right cerebellum, and left thalamus, key areas of empathy.

**Discussion:**

We conclude that patients have deficits in complex visual information processing regardless of emotional content on a behavioral level and that these deficits coincide with aberrant neural activation patterns in emotion processing networks. The right AI as an integrator of these networks plays a key role in these aberrant neural activation patterns and, thus, is a promising candidate area for neurofeedback approaches.

## 1 Introduction

In SCZ, socio-cognitive deficits are a core feature negatively affecting both treatment and prognosis (1, 2). SCZ is associated with a very high burden of disease globally, quantifiable in 13.4 million years of life lived with disability (3), an impaired ability to live independently, and social withdrawal (4). Emotion processing capabilities and empathy are of key importance to maintain social functioning, but both are often compromised in SCZ (5). Empathy is here understood as an emotional response, where the resulting emotion is similar to one’s perception of the stimulus emotion (6). This was previously coined affective sharing (7). A meta-analysis of 37 studies including self-report and performance-based measures indicated significant deficits in affective sharing in SCZ patients with a medium effect size (8). An empathic response is further determined by a more cognitive aspect, by emotion understanding, perspective taking, and emotion regulation (7). Failure in any of these components may thus impair social functioning. In fact, impaired emotion recognition may eventually lead to deficits in affective sharing (9).

Deficits in (mostly facial) emotion recognition have been a consistent finding in SCZ for decades (10–14). This has been shown for static and dynamic stimuli for inpatients (15), whereas other findings suggested that emotion recognition may remain intact when presenting dynamic faces to a heterogenous group of SCZ patients (16). It remains unclear if dynamic stimulus material has facilitating effects, but it is thought to be more ecologically valid than static stimuli (17) and should thus be further studied in patient groups.

Further underlying factors associated with emotion recognition deficits include inpatient status and antipsychotic medication, as well as higher age of both patients and controls and male gender of the control group (10, 12). SCZ symptomatology, i.e., positive and negative symptoms, may also moderate the association between emotion recognition and functional outcome (18) as well as more severe social cognitive impairments (19). Nevertheless, negative findings in a mixed group of SCZ and schizoaffective disorder patients (20) and null findings in a sample of both SCZ in- and outpatients (21) regarding the association of negative symptoms and abilities to recognize and share emotions weaken confidence in their influence. A previous meta-analysis documents that the association with symptoms varies based on which scales are used for assessment (10). Irani and colleagues found that neither inclusion of schizoaffective disorder, nor duration of illness, age at onset, and in-/outpatient status influenced emotion processing deficits in SCZ (18). To date, the association of SCZ symptom groups and social-cognitive impairments remains unclear and needs assessment with well-matched and assessed patient and control groups.

Patients with SCZ show weaker activation than HCs in a network responsible for processing facial emotion comprising the bilateral amygdalae and parahippocampal gyri, the left superior frontal gyrus, and the right middle occipital gyrus in emotion recognition tasks (22). A newer meta-analysis revealed reduced (vs. HC) activity in the right prefrontal cortex, the cingulate gyrus and insula, and in subcortical regions like the amygdala, thalamus, caudate, lentiform nucleus, and putamen as well as within the ACC and mid cingulate cortex—but increased activation (vs. HC) in the parietal cortex, a small cluster of the dorsolateral prefrontal cortex, premotor areas, and the left cuneus (23). Reduced amygdala activity has been highlighted in perceiving emotional stimuli (24). In a previous study, our group found the left thalamus, the bilateral IFG, and the cingulate cortex to be hypoactive in SCZ in emotion recognition (13). A recent meta-analysis linked emotion processing-related neural hypoactivations in SCZ of the ACC, insula, dorsolateral prefrontal cortex, and amygdala among others to be associated with social outcome of the patients.

Less is known on the neural correlates underlying affect sharing tasks in SCZ patients. Classical empathy tasks—observing another human in pain—involve dorsal ACC and AI (25, 26), both in healthy study participants and in SCZ patients. In contrast, SCZ patients also show stronger activation than HC in the left lingual gyrus and the left middle and the inferior occipital gyrus when observing images of catastrophes (27), as well as enhanced neural activity in the left insula underlying empathic reactions to comic strips (28). Affective responses to emotional descriptions of SCZ patients resulted in hypoactivation in the left superior medial frontal gyrus, the left precuneus, and the middle and posterior cingulate cortices in comparison with HC (13). Across different empathy tasks, a recent meta-analysis reported decreased activation of the right IFG in SCZ patients compared with HC (29). Differences in task design might contribute to the mixed neural findings for affective sharing.

The aim of the current study is thus an investigation into the behavioral performance and cerebral blood oxygenation level-dependent changes [BOLD] in empathy components relying on static and dynamic stimulus material. We use a simple static facial emotion recognition task, which contrasts emotion recognition and age discrimination, and a more subtle dynamic video task tapping into recognition and affective sharing in emotional and neutral stimuli. Emotion recognition capability can, thus, be compared among the different stimuli in HC and SCZ. BOLD changes of the brain are analyzed for both tasks, highlighting neural differences between simple and complex emotion processing tasks and respective alterations in SCZ patients. Furthermore, we aim to examine the interaction of SCZ symptoms, especially cognitive and negative symptoms with social-cognitive function. With mixed previous findings with regard to facial emotion recognition capabilities in SCZ, this study aims to examine whether specific groups of symptoms contribute to the deficits. Furthermore, we try to disentangle overlapping and distinct neural deficits in different aspects of empathic abilities.

We expected symptom-scores and cognitive measures to negatively predict behavioral measures both in emotion recognition and in affective sharing, and SCZ patients to show worse facial emotion discrimination performance compared with HC. Precisely, we expect a high score of negative symptoms as well as a reduced cognitive score to predict reduced emotion recognition and empathic abilities. We further expected to replicate group differences between patients and controls in the right IFG, basal ganglia, and cingulate cortices (core limbic regions) when recognizing emotions, and in the left superior medial frontal gyrus, the left precuneus, the right IFG, and the cingulate cortex when sharing an affective state.

## 2 Methods

### 2.1 Participants

All participants were fluent in German, had normal or corrected-to-normal vision, and were right-handed according to a German translation of the Edinburgh Handedness Inventory (30). In a multicenter study (DRKS00008018) from a regional network of psychiatric hospitals and consultancies, 22 patients were recruited for a randomized controlled trial (RCT, 2016–001554–18) and diagnosed by in-house psychiatrists. The inclusion criterion for the patient group was the diagnosis of SCZ (ICD 10, F20.0). Both first-episode and chronic patients were included in the sample. As part of the RCT approximately 60 days after inclusion, patients were either randomized into standard continuous antipsychotic medication vs. symptom-adapted intermittent treatment, or took part without randomization in an observational arm of the RCT. The experiments reported in this study took part before any changes were made to the medication at the timepoint of randomization. Due to the 60-day period between inclusion into the RCT and the experiments, patients were in a stable state of the disease without acute positive symptoms. There was no exclusion criteria with regard to inpatient/outpatient status. Due to high measurement duration, some patients terminated the study prematurely. Of the 22 enrolled patients, 22 concluded the emotion-recognition task, 21 the affective-sharing task, and 19 the perspective-taking task. From the HC group (total n = 51), 22 were matched (propensity score-based matching, nearest neighbor, caliper width: 0.3 log SD) to the patient sample. Matching variables were gender, age, and an estimate of verbal crystallized intelligence, measured by a German multiple-choice vocabulary test [WST] (31, 32). While sufficient German understanding was ensured, five patients were of foreign native language and hence did not complete the WST. For those patients, the mean value of the patient-group’s IQ estimates was used as a surrogate in the matching procedure. The exclusion criteria for HC was any current or previous psychiatric disorder assessed by the German SCID-IV (33).

### 2.2 Procedure

We assessed core symptomatology in SCZ applying the Scale for the Assessment of Positive Symptoms [SAPS] (34) and the Scale for the Assessment of Negative Symptoms [SANS] (35) and depressive comorbidity by the Calgary Depression Scale for Schizophrenia [CDSS] (36). The screenings took part either directly before or after the tasks. In the RCT, patients completed the Brief Assessment of Cognition in Schizophrenia [BACS], assessing different components of cognitive capacity (37). BACS was assessed as part of the enrollment procedure into the RCT (60 days prior to the reported study). All participants underwent a screening for MRI safety. This screening consisted of a questionnaire and subsequent interview checking for MRI-related contraindications. Implanted medical devices and unknown metal contamination were absolute contraindications. For metal implants (such as bone-screws or similar), manufacturer information regarding MRI compatibility was obtained.

All study procedures were in line with the standards proposed by the Declaration of Helsinki (2013). The local ethics committee of the Medical Faculty at RWTH Aachen University approved the study (EK 156/16). Oral and written informed consent was obtained from all participants. After the experiment, all participants received a 30 Euro expense compensation.

### 2.3 Tasks

**Figure 1:**
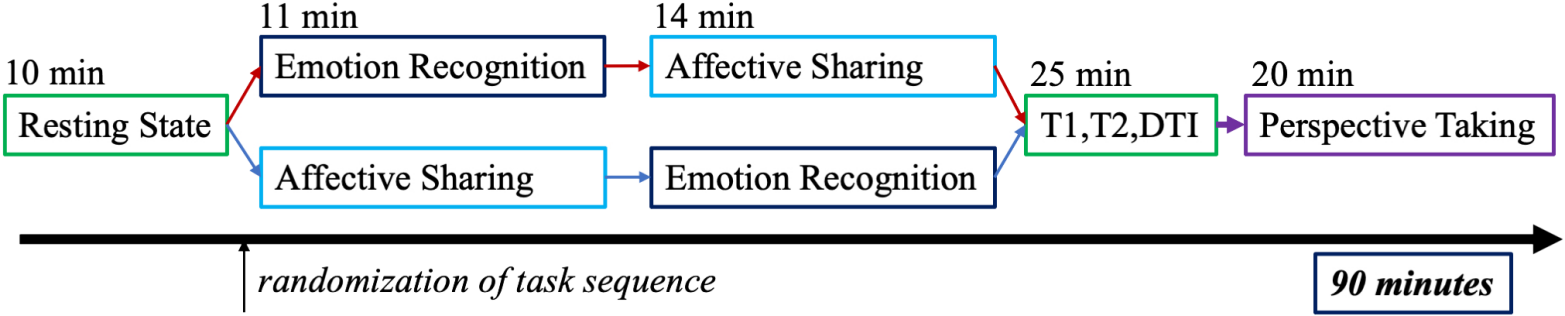
Full MRI measurement procedure with a fixed order for all participants except the emotion recognition and affective sharing task, which was carried out in an alternating order.

We applied three tasks (see **Figure 1**) testing emotion recognition, affective sharing, and perspective-taking. In the presented analyses, we focus on emotion recognition and affective sharing as a high dropout rate, due to session length, prevented an analysis of the perspective-taking-task. All tasks were delivered by Presentation software (neurobs.com, RRID: SCR_002521). Participants responded via button press using the index and middle fingers of the right hand on a LUMItouch button box (Lightwave Technologies, Richmond, Canada).

#### 2.3.1 Emotion recognition from static faces

The images for the emotion recognition task were drawn from a stimulus set developed by Gur and colleagues (38) and were validated in SCZ samples (13, 39). A total of 60 color-photographs of Caucasian faces of different age and sex, depicting either a neutral expression or one of the five basic emotions (sadness, happiness, fear, anger, disgust), were presented for a maximum of 4 s. Selecting between two options, participants should identify the presented emotion or, as a control condition, age decade (twenties–seventies). A central fixation cross on a beige screen was shown after button press varying from 4.25 to 10 s as interstimulus interval (ISI). Faces were presented in a pseudorandomized order fixed across participants whereby half of the trials were allocated to the emotion recognition, the other half to the age discrimination condition. The task lasted approximately eleven minutes.

#### 2.3.2 Affective sharing and emotion recognition from dynamic faces

In this task, participants watched 32 short video clips (mean 10.3 s) of Caucasian actors in close-up portrait. The videos consisted of short self-related narrations of the actors with emotional content (four scenes each for sadness, happiness, fear, disgust) or neutral content (16 in total). In the neutral videos, actors were filmed narrating stories without emotional content. Highlighting the visual emotional information and relying on unisensory stimulation only, sound was muted for both tasks. Participants, thus, relied solely on the visual facial information. As the task aimed at evoking the sharing of the emotions recognized in the actors, participants were instructed to imagine themselves in the situation of a close relative or friend of the actor. A personal relationship should facilitate an empathic response. This approach has been used and discussed by our research group before (16, 40) and is aimed at preventing the missing relation of actor and participant from blocking emotional involvement in the participants. Moving a cursor on a visual analogue scale with five options, after each video, participants indicated what emotion the actor felt in the video and what emotion the participants felt themselves. After 4.5 s, the cursor position was logged as the chosen answer followed by a fixation cross (ISI of 4.8 s–8 s). The task lasted approximately 14 min.

### 2.4 Data analysis

#### 2.4.1 Behavioral data analysis

SPSS 25 (IBM, RRID: SCR_002865) was used for the statistical analysis of behavioral data. Threshold of significance was set to *p < 0.05*. Bonferroni correction was applied in case of multiple comparisons. The percentage of correct answers [%correct] was used as primary outcome parameter for task performance in both tasks. In the emotion recognition task, mean response time [RT] was tested as further parameter. Responses given during the interstimulus interval were not included into the analysis. %correct and RT were analyzed using ANOVAs. GROUP (SCZ/HC) was used as the between-subject factor, and CONDITION (emotion/age for emotion recognition task, emotion/neutral for affective sharing task) was defined as the within-subject factor. In the affective sharing task, secondary scores included an *empathy score* and *correspondence score. Empathy* was calculated as the proportion of congruent answers on self and actor’s emotion among all correctly recognized emotions. *Correspondence* was calculated as the percentage of congruently selected emotions independent of the correctness of the chosen emotion. Furthermore, the *share* of participants’ emotions matching the emotion portrayed in the video was calculated, regardless of congruency with the emotion recognized in the actor. If data were normally distributed group-means of these secondary scores were compared using independent sample t-tests. Mann–Whitney U tests were used as non-parametric analyses in case of violations against normality assumptions. The level of significance was adjusted to *p < 0.05/3 = 0.0167*. Pearson correlation was used to test for the association between emotion recognition from static and dynamic stimuli.

As explorative analysis, a stepwise regression model was applied to test whether psychopathology (global scores of SAPS and SANS, global score of CDSS), cognitive estimates (BACS composite score, WST), and age predict the behavioral results. Behavioral results of the emotion condition (see above) were entered as dependent variables. Applying Bonferroni correction, the threshold of significance for the regression analyses was set to *p < 0.05/6 = 0.0083*.

#### 2.4.2 fMRI acquisition and preprocessing

MRI measurements were performed at the 3 Tesla Siemens MAGNETOM Prisma MRI-scanner (Siemens AG, München, Germany) at the Department of Psychiatry, Psychotherapy and Psychosomatics in the University Hospital of Aachen. Functional images were acquired using echo planar imaging sequences sensitive to BOLD. There were 34 axial slices of 64 × 64, 3 × 3 × 3 mm³ voxel resulting in a field of view of 192 × 192 mm² (0.465 mm gap, TR/TE 2,000/28 ms, flip angle: 77°). A total of 300 and 420 images were acquired for the emotion recognition and affective sharing task, respectively. Furthermore, an anatomical measurement was acquired (T1 weighted 3d image, magnetization prepared rapid acquisition gradient echo image, 1 mm³ voxel size, TR = 2,000 ms, TE = 3.03 ms, TI = 900 ms, matrix = 256 × 256, 176 slices, flip angle = 9°, duration = 4 min). Movement parameters were screened for all participants in six directions (x, y, z, roll, pitch). Participants exceeding 4 mm of head motion in any dimension were excluded from the analysis leading to the following sample size: emotion recognition, 20SCZ vs. 21HC; affective sharing 19SCZ vs. 20HC.

#### 2.4.3 fMRI data analysis

Functional images were processed with SPM12 (Wellcome Department of Cognitive Neurology, London, United Kingdom, RRID: SCR_007037) implemented in Matlab 2018b (MathWorks Inc., Natick, Massachusetts, USA, RRID: SCR_001622). Preprocessing included realignment, co-registration to the anatomical image, normalization into Montreal Neurologic Institute [MNI] image space, and spatial smoothing. Unified segmentation (41) was applied in order to assess normalization parameters for the transformation to MNI space. An isotropic gaussian kernel with 6-mm full-width-a-half-maximum was used for spatial smoothing.

On the individual level (first-level statistics), six motion parameters and task stimulus functions were modeled comprising emotion trials (single regressor across all emotions) and control trials (single regressor for age estimation trials in emotion recognition and neutral trials in affective sharing) for the emotion recognition and affective sharing task, respectively. Both tasks were analyzed using a box-car design. In first-level analysis, these regressors were contrasted against fixation cross. The stimulus duration was 9 s–11 s in the affective sharing task and a maximum of 4 s in the emotion recognition task. The stimulus functions were convolved with the canonical hemodynamic response function. Furthermore, the intercept for the complete scanning session modeled the mean of the time series. A high-pass filter of 7.81 mHz (cutoff period of 128 s) was applied to remove low-frequency drifts. Parameter estimates were obtained after accounting for temporal autocorrelations (AR1).

On the second level, a full-factorial design was applied for each task. Condition (emotional stimuli vs. control stimuli) was used as a within-subject factor and group (SCZ vs. HC) as a between-subject factor. We report F-contrasts assessing the interaction of group and condition and directed t-contrasts comparing the main effects of group and conditions. Results were obtained using a cluster-defining threshold of *p <.001* for an FWE cluster-level correction at *p <.05*. *A* conjunction analysis of the emotion and age (emotion recognition task) or neutral (affective sharing task) contrasts was conducted. The anatomy toolbox (42) (RRID: SCR_013273) was used to provide information on the localization of significant clusters. MRIcroGL (https://www.nitrc.org/projects/mricrogl/, RRID: SCR_002403) was used to create all images displaying fMRI results.

## 3 Results

### 3.1 Study sample

Sample characteristics and symptom scores of the SCZ group are depicted in **Table 1**. Premorbid intelligence estimation (*t = .139, p = .850*), mean age (*Z = −1.410; p = .159*), and gender (Chi-Square = .140, p = .709) of participants did not differ significantly between groups.

**Table 1:** Study sample: psychiatric status and matching criteria of study sample.

Characteristics	SCZ (n = 22)	HC (n = 22)	*p*
Male, n Female, n Age (mean ± SD)	17 5 34.4 ± 11.5	18 4 30.2 ± 10.9	.709 .159
Premorbid intelligence			
WST (mean ± SD)	101.1 ± 9.9	101.5 ± 11.8	.850
Psychopathology			
SAPS (mean ± SD) SANS (mean ± SD) CDSS (mean ± SD)	5.0 ± 4.3 7.9 ± 6.9 4.4 ± 5.3		

### 3.2 Behavioral results

The analysis of RT in the emotion recognition task (**Table 2**) differed significantly between groups and conditions. SCZ patients responded slower than HC and both groups took longer to estimate age compared with the emotion. Paired *post-hoc* tests on the interaction of group and condition showed that SCZ patients were only slower than HC in the age condition, but not in the emotion condition (**Table 3**). Note that the descriptives, however, show the same tendency in the emotion condition. A similar pattern emerged for %correct with both groups showing less accuracy for age compared with emotion trials. There was no significant effect of group, nor a significant interaction of group and condition (**Table 3**). In the affective empathy task, there was neither a main effect of group or condition, nor was there a significant interaction.

**Table 2:** Behavioral results: primary and secondary behavioral outcome parameters.

Task	SCZ	HC
Emotion recognition	*n = 22*	*n = 22*
RT emotion stimuli (mean [ms] ± SD) RT emotion stimuli (median [ms]; range) RT age stimuli (mean [ms] ± SD) RT age stimuli (median [ms]; range) RT both (mean [ms] ± SD) RT both (median [ms]; range)% correct emotion recognition (mean [%] ± SD) % correct emotion recognition (median [%]; range) % correct age discrimination (mean [%] ± SD) % correct age discrimination (median [%]; range) % correct both (mean [%] ± SD) % correct both (median [%]; range)	2065.7 ± 317.7 2033.3; 1074.5 2302.5 ± 254.2 2277.4; 926.9 2174.4 ± 278.8 2157.5; 991.6 88.0 ± 12.6 93.1; 49.3 75.9 ± 11.0 76.7; 50.0 82.0 ± 11.2 84.2; 49.7	1888.2 ± 287.9 1843.1; 1156.6 1993.0 ± 360.4 1961.3; 1347.6 1936.1 ± 310.2 1955.4; 1240.8 91.1 ± 6.2 93.1; 26.7 82.2 ± 7.0 80.0; 28.8 86.7 ± 5.4 87.5; 20.0
Affective sharing	*n = 21*	*n = 21*
Empathy score (mean [%] ± SD) Empathy score (median [%]; range) Correspondence score (mean [%] ± SD) Correspondence score (median [%]; range) % correct emotional stimuli (mean [%] ± SD) % correct emotional stimuli (median [%]; range) % correct neutral stimuli (mean [%] ± SD) % correct neutral stimuli (median [%]; range) Share matched emotional stimuli (mean [%] ± SD) Share matched emotional stimuli (median [%]; range)	41.6 ± 31.9 40.0; 100 40.2 ± 27.3 31.3; 87.5 66.1 ± 19.6 62.5; 75.0 69.6 ± 15.6 75.0; 68.8 .295 ± .218 .313; .750	56.2 ± 28.3 61.5; 100 53.9 ± 28.1 56.3; 93.8 77.7 ± 13.3 81.3; 50.0 71.7 ± 15.0 75.0; 56.3 .438 ± .223 .500; .750

**Table 3:** Behavioral results.

Task	*F*	*dF*	*p*
Emotion recognition RT			
Group (SCZ vs. HC)	7.478	1	.009*
Condition (emotion vs. age)	43.703	1	<.001*
Group*condition	6.536	1	.014*
Emotion: SCZ vs. HC	3.769	1	.059
Age: SCZ vs. HC	10.841	1	.002*
SCZ: emotion vs. age	42.021	1	<.001*
HC: emotion vs. age	8.218	1	.006*
Emotion recognition %correct			
Group (SCZ vs. HC)	3.131	1	.084
Condition (emotion vs. age)	81.341	1	<.001*
Group*condition	1.770	1	.191
Affective sharing %correct			
Group (SCZ vs. HC)	3.119	1	.085
Condition (emotion vs. neutral)	.149	1	.702
Group*condition	2.378	1	.131

Test statistics. Significant results are marked with an asterisk. Threshold of significance was set to p <.05.

No significant differences emerged in the comparison of groups in secondary scores in the affective sharing task. Recognition of Emotion in static and dynamic stimuli correlated positively (Pearson correlation coefficient:.558, p ≤.001).

#### 3.2.1 Stepwise regression analysis in SCZ

**Figure 2:**
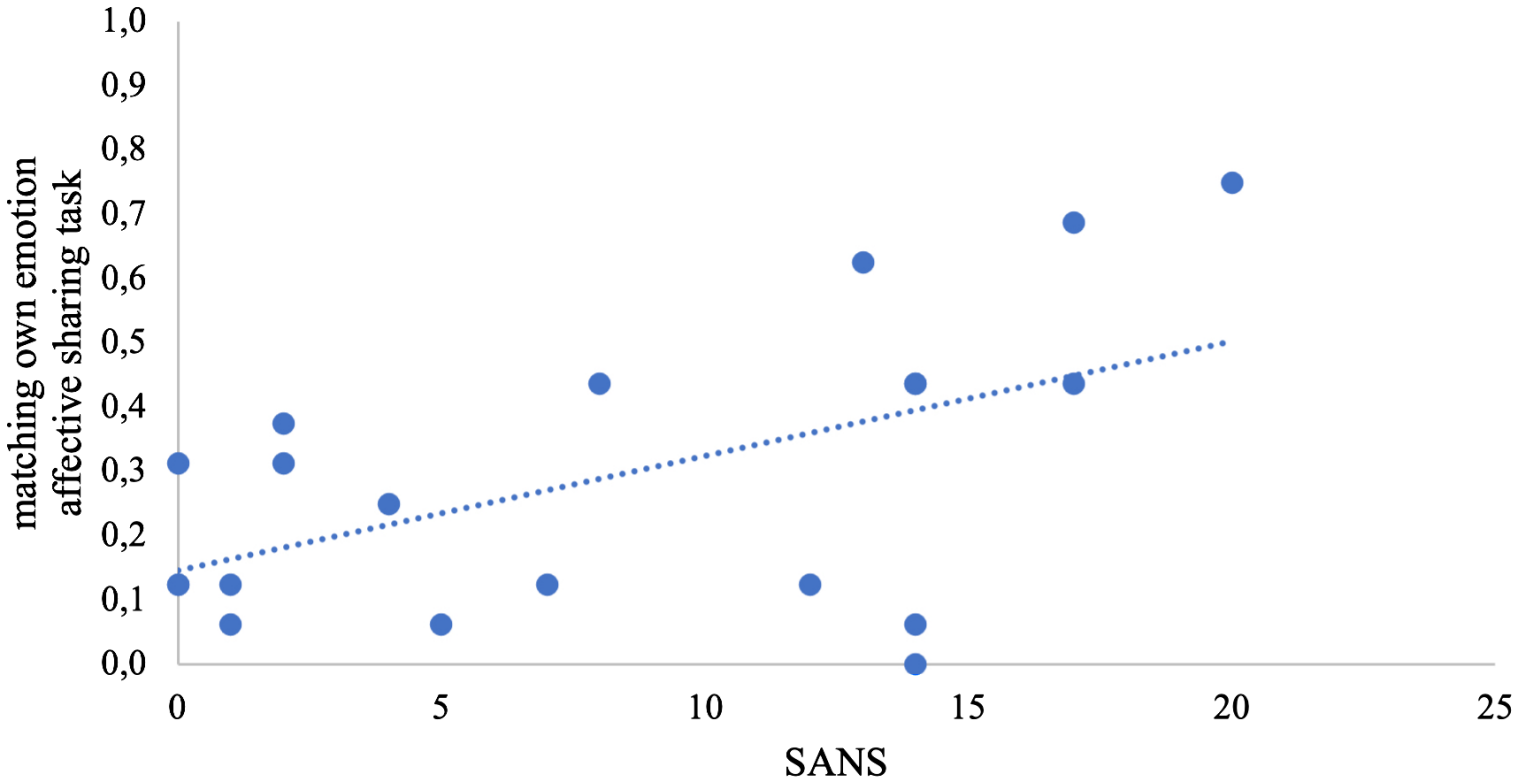
Scatterplot of the stepwise regression model. The share of matching own emotions is depicted in the y-axis; the total score of negative symptoms (SANS) is depicted in the x-axis.

Both in the emotion recognition task and in the affective sharing task, the WST score alone best explained variance of %correct but did not survive correction for multiple comparisons. In the affective sharing task, only the regression model with the SANS score as predictor for the number of matching emotions of actor and participant was significant after applying Bonferroni corrections (**Table 4**, **Figure 2**).

**Table 4:** Stepwise regression analyses: significant regressions are marked with an asterisk.

Dependent variables	Variables entered	*Correlation coefficient*	*R² change*	*p*
Emotion recognition				
% correct emotion recognition	WST	.557	.310	.020
Affective sharing				
Empathy score % correct emotional stimuli actor Correspondence score emotion Share matched emotional stimuli	SANS WST SANS SANS	.505 .592 .584 .677	.255 .350 .341 .458	.046 .016 .018 .004*

Threshold of significance was set to p < 0.05/6 = 0.0083.

### 3.3 fMRI results

**Figure 3:**
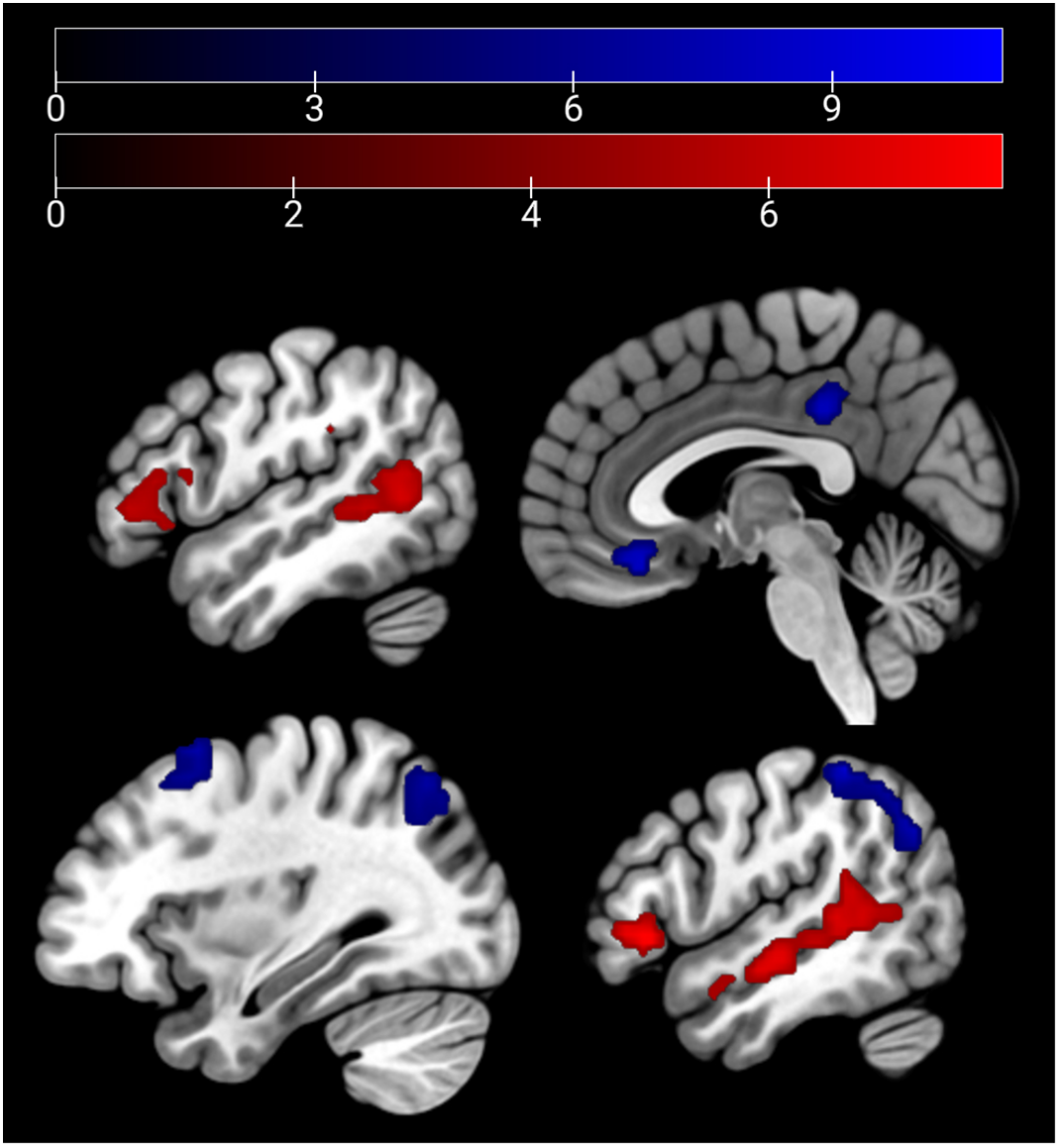
Main effect of condition: emotion > age depicted in red (bilateral middle temporal gyrus, supra-marginal and IFG) and age > emo in blue (right lateral occipital cortex, right superior and middle frontal gyrus, frontal medial cortex, and posterior cingulate cortex) at p <.05 peak-level FWE corrected. Sagittal slices −51, 3, 33, and 51 are depicted.

In the emotion recognition task, the emotion condition (across groups) elicited a stronger (compared with age estimation condition) activation in the bilateral middle temporal gyrus, supramarginal gyrus, and the bilateral IFG. For age estimation, a pattern comprising the right lateral occipital cortex, the right superior and middle frontal gyrus, and the frontal medial cortex and posterior cingulate cortex (**Figure 3**).

**Figure 4:**
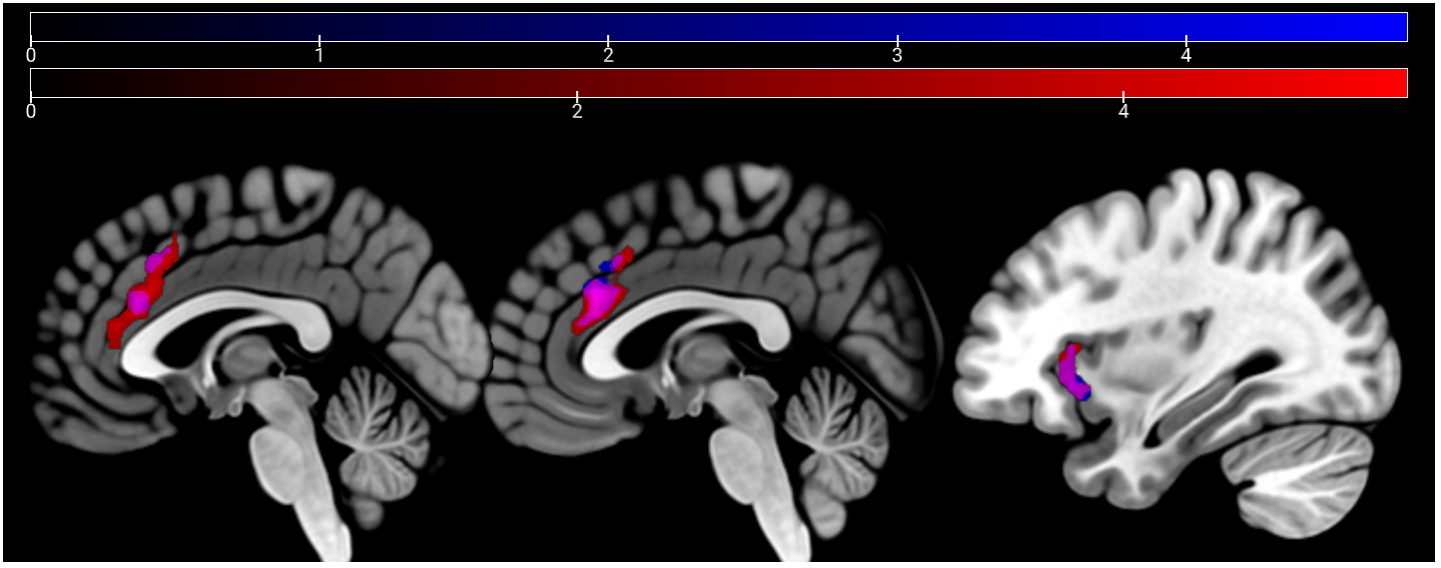
Emotion and age recognition HC > SCZ contrasts: emotion recognition trials are depicted in red and age estimation trials in blue at p <.05 cluster-level FWE corrected. Overlapping areas are colored violet (bilateral ACC and right AI). Sagittal slices −2, 2, and 33 are depicted.

**Figure 5:**
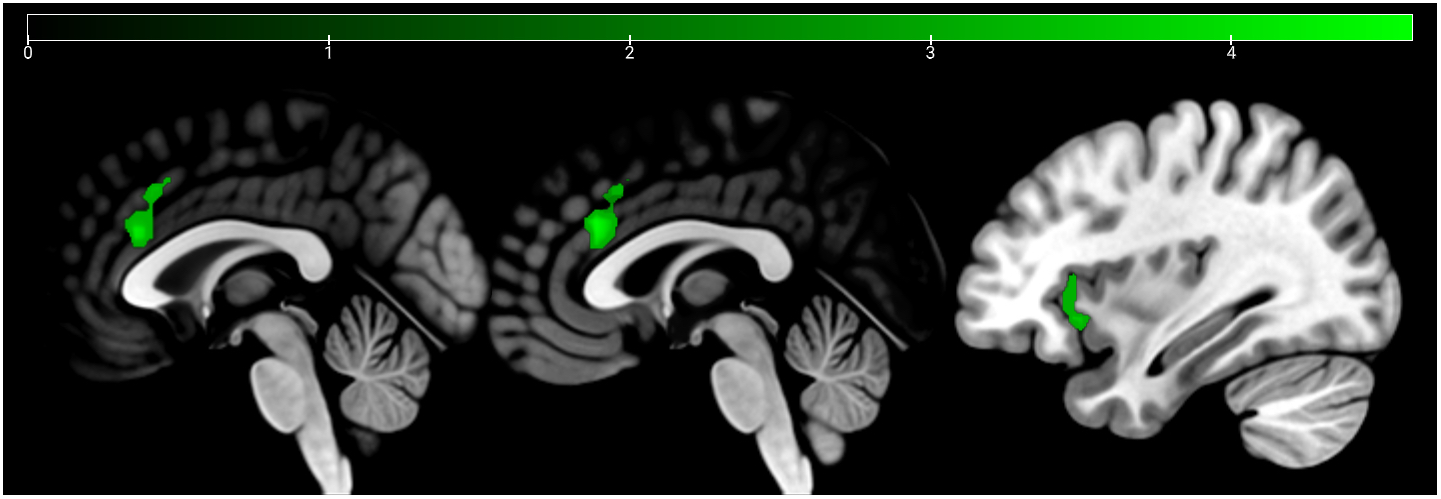
Conjunction analysis (emotion and age contrast) HC > SCZ. Cluster marked in green (bilateral ACC and right AI) at p <.05 cluster-level FWE corrected. Sagittal slices −1, 1, and 34 are depicted.

HC showed a stronger activation of the bilateral ACC and the right AI than SCZ patients in emotion recognition trials. During age estimation, the same pattern emerged (**Table 5**, **Figure 4** *)*. A conjunction analysis including age and emotion contrasts showed a similar pattern with activation of the bilateral ACC and right AI (**Figure 5**). No significant cluster was observed for the reverse contrast (SCZ > HC).

**Table 5:** Emotion recognition HC > SCZ: coordinates that have been listed as area not specified by the anatomy toolbox are marked with an asterisk.

*p FWE* *clusterlevel*	Number of voxels	*t* *peaklevel*	*p(unc.)* *peaklevel*	MNI coordinates			Anatomy toolbox area peaklevel
				x	y	z	
Emotion recognition							
<0.001	140	5.13	<0.001	0	29	26	L ACC (BA 32)
		4.30	<0.001	-6	35	8	L ACC (BA 24)
		4.19	<0.001	0	20	41	L superior medial gyrus (BA 8)
0.001	95	4.59	<0.001	30	23	-4	R insula* (BA 13)
		4.36	<0.001	45	23	8	R IFG (BA 45)
		3.96	<0.001	39	20	-10	R insula (BA 13)
Age estimation							
0.020	56	4.87	<0.001	30	23	-4	R insula* (BA 13)
		4.37	<0.001	39	20	-10	R insula (BA 13)
		3.98	<0.001	45	23	8	R IFG (BA 45)
0.013	62	4.76	<0.001	3	29	29	R MCC (BA 8)
		3.67	<0.001	0	20	41	L superior medial gyrus (BA 8)
		3.63	<0.001	6	32	17	R ACC (BA 32)

Brodmann areas [BA] are listed when possible.

**Figure 6:**
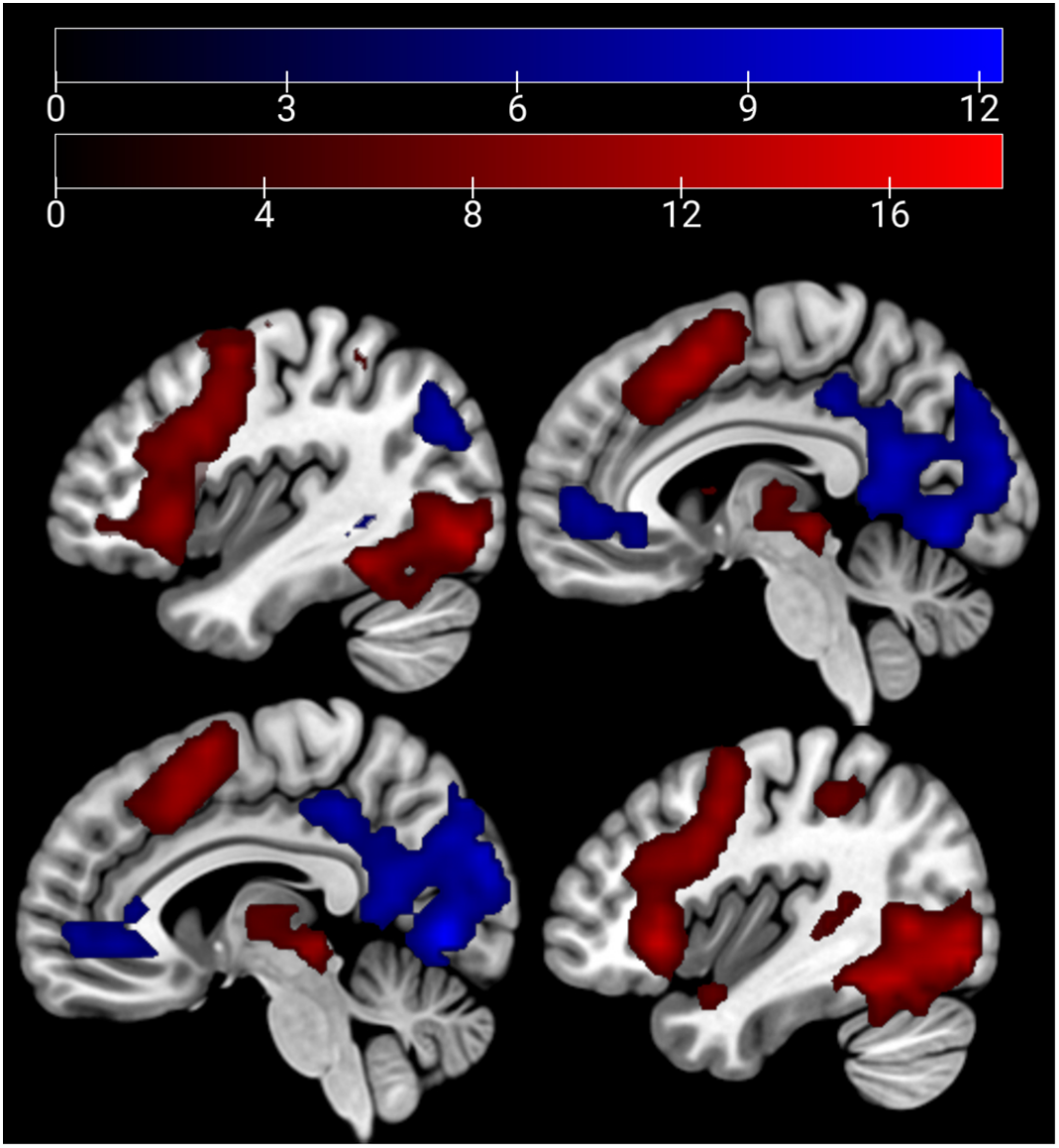
Main effect of condition: emotion > neutral depicted in red (bilateral occipital poles, insulae, paracingulate gyri, superior parietal lobules, and caudate nuclei) and neutral > emo in blue (bilateral occipital poles, lingual gyri, paracingulate cortices, and lateral occipital cortices) at p <.05 peak-level FWE corrected. Sagittal slices −40, −6, 6, and 40 are depicted.

**Figure 7:**
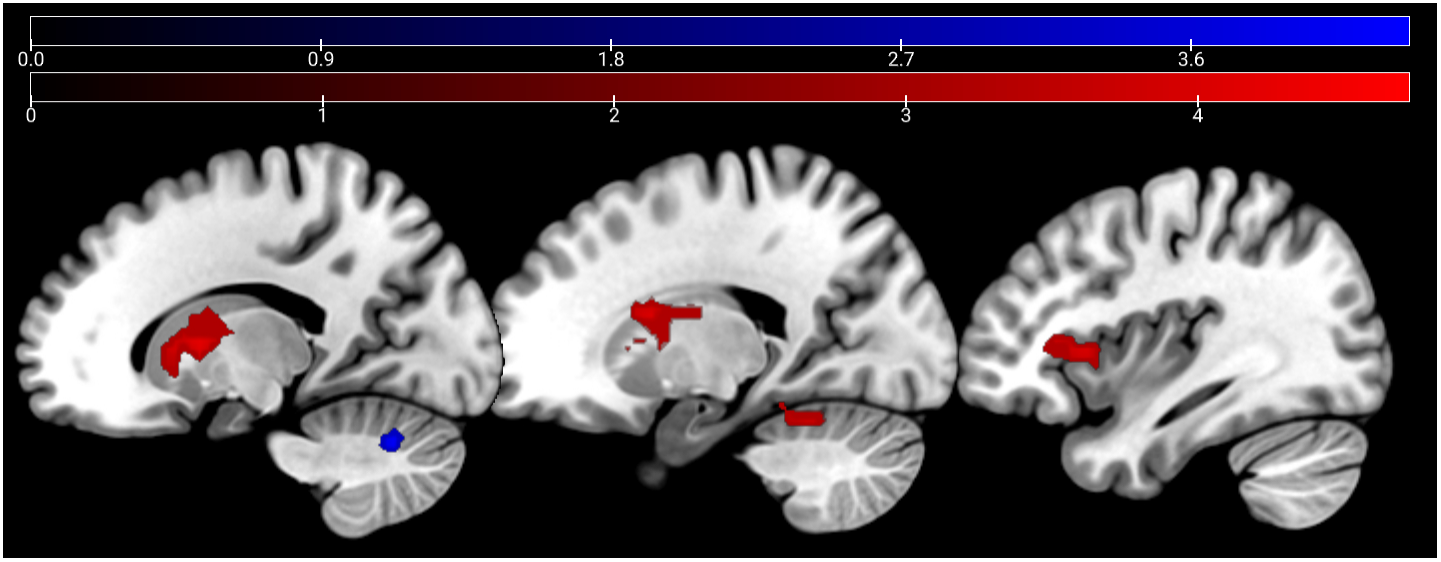
Affective sharing: HC > SCZ contrasts for emotional trials are depicted in red (bilateral caudate nucleus, right AI, IFG, cerebellum, and the left thalamus) and neutral trials in blue (left cerebellum) at p <.05 cluster-level FWE corrected. Sagittal slices −16, 19, and 37 are depicted.

In the affective sharing task, emotional trials (vs. neutral trials) lead to a stronger activation of the bilateral occipital poles, insulae, paracingulate gyri, superior parietal lobules, and caudate nuclei across groups. Neutral stimuli similarly (vs. emotional trials) activated areas of the bilateral occipital poles, lingual gyri, paracingulate cortices, and lateral occipital cortices (**Figure 6**). The HC group showed a stronger activation than the SCZ group in the left and right caudate nucleus, the right AI and IFG, the right cerebellum, and the left thalamus during emotional trials. During neutral trials, HC showed a stronger activation of the left cerebellum (Lobule VI) (**Table 6**, **Figure 7** *).* No significant clusters were found for the reverse group contrast. A conjunction analysis of emotional and neutral stimuli revealed no clusters that survived cluster-level FWE correction.

**Table 6:** Affective sharing HC > SCZ: coordinates that have been listed as area not specified by the anatomy toolbox are marked with an asterisk.

*p FWE* *clusterlevel*	number of voxels	*T* *peaklevel*	*p(unc.)* *peaklevel*	MNI coordinates			Anatomy toolbox area peaklevel
				x	y	z	
Emotional stimuli							
<0.001	128	4.73	<0.001	15	5	17	R caudate nucleus
		4.02	<0.001	3	−7	11	R temporal thalamus
		3.80	<0.001	12	11	8	R caudate nucleus
0.001	119	4.42	<0.001	−15	8	8	L caudate nucleus*
		4.32	<0.001	−15	2	17	L caudate nucleus
		4.19	<0.001	−18	17	5	L caudate nucleus
0.002	102	4.16	<0.001	36	17	5	R insula (BA 13)
		3.87	<0.001	36	29	8	R IFG (BA 45)
		3.84	<0.001	27	17	5	R putamen
0.024	59	4.13	<0.001	27	−40	−25	R lobule IV/V
		4.00	<0.001	18	−49	−22	R lobule IV/V
		3.88	<0.001	3	−49	−1	R lobule IV/V
Control stimuli							
0.012	70	4.42	<0.001	−24	−61	−31	L lobule VI
		4.25	<0.001	−15	−67	−28	L lobule VI
		4.09	<0.001	−27	−52	−34	L lobule VI

BA are listed when possible.

## 4 Discussion

Comparing the empathy components emotion recognition and affective sharing between SCZ patients and HC, we identified deviations on a behavioral and neural level in patients. Against our expectations, behavioral results failed to support a specific impairment of identification or sharing of emotions. Descriptive statistics only indicate a trend into a deficit of SCZ patients.

### 4.1 Generalized cognitive perceptual deficits in schizophrenia

We observed reduced age discrimination performance, but no significant group differences in recognizing emotions from static facial displays. Whether facial perception deficits in SCZ are emotion-specific or general has been a long debate (43). Deficits even in only a subset of basic emotions (mostly negative or aversive) have been reported by multiple studies (44–46). At the same time, generalized deficits in facial perception have also been reported (47, 48). Our results may support these generalized deficits in the perception and processing of facial information. One explanation is that reduced performance in SCZ is more prominent in complex tasks, an observation that has been made before (47). Accordingly, a more complex task—rating the correct age decade—compared with a more intuitive task—recognizing the emotion from a face—may provoke the more pronounced deficits in performance. In other words, only two possible alternative emotions were displayed as answers, rendering the decision pretty easy (but see (13, 21). A second explanation is that the small sample size and high heterogeneity of performance might have prevented descriptive group differences in the emotion condition for both static and dynamic stimuli to reach significance. Also, the low degree of negative symptoms present in the study sample might have added to the statistical behavioral equality in the group comparisons. Mostly, negative symptoms in general are linked to emotion processing deficits (49). In addition, there is specific evidence for affective flattening in contrast to other negative symptoms to predict emotion processing performance (50). Future research should try to address how patients differing in clinical characteristics on the SCZ spectrum and, thus, differing in the degree of specific symptoms, can be differentiated with regard to emotion processing performance.

Recently, a line of research also started to differentiate between different decoding processes involved in so-called invariant (here: the age discrimination task) and interchangeable (here: the emotion recognition task) aspects involved in perceiving facial characteristics (51) and, more importantly, that these processes might be differently affected in SCZ. In our study, we did find evidence only for invariant (age) features of face perception in patients with SCZ whereas other studies found deficits for both invariant and interchangeable aspects in complex facial decoding (48). However, as the *a priori* focus was not specifically on investigating this question, the results should be interpreted with caution and followed up in future studies.

Finally, while several studies point toward the finding that facial expressions are more difficult to categorize in dynamic than in static stimuli for SCZ patients (17), we could not clearly find evidence for this. While a direct comparison (a task in which the same static and dynamic stimuli was used) was not possible, we can state, however, that deficits in emotion recognition did not manifest using dynamic material in the affective sharing task. Precision in recognition correlated in static and dynamic stimulus results. In a previous study, we found emotion recognition and empathy impairments in SCZ patients when speech content did not match prosody and facial expression of the dynamic information (16); however, in this previous study, a different task design was used and the condition in which we had neutralized emotional facial expression (with remaining emotionality in speech content and prosody) did not significantly reduce the affective sharing of the patients compared with the controls. Overall, a large heterogeneity of behavior was visible in the patient group in dynamic stimuli, which might have prevented the descriptively visible group differences to reach significance.

### 4.2 Negativity bias leading to increased affect sharing of negative emotions

Also, against our hypothesis, negative symptoms (SANS) correlated with the percentage of shared emotions, which means that patients with worse negative symptom scores shared the emotion of the depicted other to a higher degree than patients with mild negative symptoms. At first sight, this may be surprising as typically, negative and positive symptoms are associated with decreased empathy measures (13, 20). However, when looking at our stimulus material, 75% off the portrayed emotions were negative (sadness, disgust, anger). It is known for other psychiatric diseases, such as depression, that negative facial expressions are recognized more successfully due to a negativity bias (52). Although negative symptoms are not comparable with depression, there might be a potential sharing bias for negative emotions with higher negative symptoms. This might have contributed to this correlative finding in SCZ. A different explanatory approach to this surprising result is reduced self-other distinction that has been reported as high emotion contagion and personal distress in some SCZ samples before (53, 54). The current study, conceptualizing empathy as equivalence in self and other’s emotion, supports a higher degree of shared emotions for patients with disturbed self-other distinction and high emotional contagion.

### 4.3 Hypoactivation in limbic empathy-related network in schizophrenia

Although behavioral analysis did not show specific group deficits in emotion processing, we analyzed the neural activity during the completion of the tasks. We argue that an insight into neural activity during tasks tapping into different areas of social cognition might show aberrant neural activity in SCZ contributing to deficits in social cognition, even if the task design and/or difficulty failed to uncover the deficits on a behavioral level.

For both static and dynamic emotional face processing, SCZ patients show reduced activation of the right anterior insula. The insular cortex has been shown to activate in affective perceptual empathy (55). Furthermore, the insular cortex has been shown to activate in emotional interoceptive processes (56) and one’s own reactions to recognizing others’ emotions (57). A large meta-analysis of fMRI and lesion data points out the AI is an integrator of a network of interoception, emotion processing, and social cognition (58). This function could hint at the AI cortex as a key area for neurofeedback or neuromodulation techniques in patients suffering reduces sociocognitive abilities. Early data on AI upregulation through neurofeedback show promising results for empathic abilities (59), and both neurofeedback and neurostimulation have been shown to be effective in SCZ (60, 61). Furthermore, a better understanding of neural circuits can aid focused psychotherapies aiming to enhance neuroplasticity in social cognitive networks, as a recent review notes (2). Well-guided functional neuroimaging might, thus, also help in assessing therapeutic efficacy of novel tools.

Both MRI tasks revealed reduced activation of an extended network of brain areas in patients, which matches previous findings. Hypoactivation during emotion recognition in the left ACC and right AI in SCZ supports previous empirical findings (13, 22) and matches meta-analyses (23, 24). The ACC and insular cortex have been reported before to be associated with the processing of one’s own (62) and other peoples’ pain (63). The ACC is related to general emotion processing (64). In our sample, the same AI and ACC pattern emerged for age estimation. This shared activation deficit might hint at our hypothesis of a non-emotion-specific deficit in face perception. As stated above, the AI is known to activate in interoceptive emotional processes; the overlapping activation in both tasks might, thus, represent a subjective emotional reaction to the emotion portrayed in the stimulus material regardless of the task’s focus as well.

Contrasting this conceptualization, the role of the ACC and insula also extends toward non-emotion specific functions. The insula has been linked to network switching, acting in bottom-up detection of salient events in interaction with the anterior cingulate cortex, which is linked to motor regions enabling behavioral response to such salient events (65). Another review links the ACC and insula in connection with prefrontal cortical areas to hot executive function involved in theory of mind and social cognition, as opposed to purely cognitive or “cold” executive function it attributes to a network spanning the ACC, hippocampus, and prefrontal cortical areas (66). Our data hint at disturbances in the interaction in SCZ for both hot and cold executive function. Alternatively, the shared pattern in hypoactivation might result from the stimulus material being similar, leading to emotion-related processing also in the age discrimination processing. Importantly, previous research links dysfunction of the white-matter tracts of the cingulate cortex to chronic SCZ but highlights its importance in the psychotic states, rather than cognitive function (67). More detailed research into ACC and insula interaction will be needed to disentangle its dysfunction in different symptoms or aspects of SCZ. During video clip presentation in the affective sharing task, patients showed reduced activation of the left and right caudate nucleus, the right AI and IFG, the right cerebellum (lobules IV and V), and the left thalamus. These areas recruited differently by patients and controls mostly relate to cognitive empathic processes (i.e., theory of mind tasks), as concluded in a recent meta-analysis (23). An incisive distinction of brain areas involved in cognitive and affective empathic processes might not be possible.

Previous research on the neural pathways implicated in silent lip-reading (68) suggests a link between the IFG hypoactivation detected in our study with the perception of the presented silent speaking faces. It is the left IFG, however, that is predominantly reported in lip-reading, whereas in our study hypoactivation in the right IFG was observed during emotional narration. Interestingly, this hypoactivation was not present for neutral narration. We thus conclude that the emotional component was important for the activation difference. The IFG is also part of a robust network active in auditory verbal hallucinations that are often present in patients with SCZ (69). Our patient sample displayed only mild to no positive symptoms as rated by the SAPS, yet it is unclear if IFG hypoactivation may be linked to auditory hallucinations. As the activation differences were only found during the presentation of emotional stimuli and have been linked to social cognitive deficits in SCZ (23), we argue that much evidence supports an interpretation linking altered neural activations to social-cognitive deficits in SCZ.

The cerebellar lobules IV and VI have been linked to mentalizing networks that comprise prefrontal areas as well as the insula, among others (70). While watching video clips with neutral mimics, only a small cluster in the cerebellum was found to be hypoactive in SCZ. Also for neutral mimics, mentalizing abilities play a role, which might explain the shared hypoactivation in the two conditions. The cerebellum has also been implicated in finger tapping (71), and button presses could have led to this effect (71). The descriptives of the behavioral data show an intact recognition of neutral facial mimics and a reduced (although below threshold of significance) recognition of emotional mimics. The imaging data support this seemingly impeded ability in recognition of dynamic emotional mimics.

### 4.4 Strength and limitations

Our study used both static and dynamic stimulus material of facial emotion expression, and tasks assessing emotional and non-emotional information, in order to thoroughly test for empathy component deficits in SCZ. Although emotion recognition is an important aspect enabling empathy, corresponding emotional sense or concern for the emotion demonstrator was inferred from the responses of the participant and was not directly tested; thus, not all aspects of empathy were addressed in this study. Previous research has raised concerns whether actor-based tasks are ecologically valid in examining social-cognitive function (53). The largest limitation of our study is the small sample size reducing the statistical power of the analysis (72). The small sample size prevented us from analyzing subgroups in the heterogenous (chronic and first episode) SCZ sample. Yet, our sample largely reflects the norm as SCZ is characterized by symptom and progression heterogeneity. In the emotion-recognition task, the age estimation was not well-matched regarding difficulty to the emotion-recognition condition. The very basic assessment of cognitive function, especially for the healthy control group, prevented a more detailed analysis of the influence of cognitive function on emotion recognition and affective sharing. Furthermore, the samples’ stable stage of the disease prevented subgroup comparisons with acutely symptomatic patients. Previous enrollment into an RCT might have biased the patient sample, as potentially more stable and highly motivated patients agreed to take part in the RCT. Current and history of psychotherapy, counseling, and use of other non-pharmaceutical therapeutic tools were not assessed, preventing an analysis of therapy-specific effects. The study sample included mostly male Caucasians preventing the analysis of gender or culturally related differences.

A direct comparison of concomitant brain activity in static and dynamic emotion recognition was not possible due to a task design in the affective sharing task. The differences in neural activations during social-cognitive tasks were not accompanied by significant differences in behavior between the two groups. We argue that the aberrant neural pathways might hint at differences that contribute to the social-cognitive deficits SCZ patients encounter in the course of their disease. Future research will have to focus on matching task designs that both elicit measurable differences in behavior and differences in neural activity patterns.

## 5 Conclusion

Based on reduced performance in more difficult categorization tasks, we conclude that SCZ patients have difficulties with complex information processing, which is not specifically related to emotion processing. Patients responded slower to facial processing tasks. *Post-hoc* tests revealed a significantly slower response only to age discrimination tasks. No behavioral deficits in the recognition and sharing of emotion in dynamic stimuli were found. Furthermore, a potential negativity bias in SCZ patients with more negative symptoms may have actually led to performance increase in the affective sharing task, leading to increased sensitivity to sharing negative emotions of the other. The deviating association of neural activation and behavioral patterns might indicate altered processes counteracting deficits in information processing with a crucial role for the right anterior insula as integrator of emotional and interoceptive brain networks.

## Data availability statement

The raw data supporting the conclusions of this article will be made available by the authors, without undue reservation.

## Ethics statement

The studies involving humans were approved by the Medical Faculty at RWTH Aachen University (EK 156/16). The studies were conducted in accordance with the local legislation and institutional requirements. Written informed consent for participation in this study was provided by the participants or the participant’s legal guardian/next of kin.

## Author contributions

SK: Data curation, Formal analysis, Investigation, Writing – original draft, Visualization, Writing – review & editing. DL: Conceptualization, Formal analysis, Investigation, Methodology, Writing – review & editing. LW: Formal analysis, Methodology, Software, Writing – review & editing. CR: Formal analysis, Methodology, Software, Writing – review & editing. TK: Software, Writing – review & editing. KM: Project administration, Writing – review & editing. FS: Funding acquisition, Project administration, Writing – review & editing. BD: Project administration, Writing – review & editing. UH: Conceptualization, Funding acquisition, Methodology, Project administration, Resources, Supervision, Writing – review & editing.
